# Effects of Surfactants on the Preparation of Nanocellulose-PLA Composites

**DOI:** 10.3390/bioengineering4040091

**Published:** 2017-11-17

**Authors:** Kirsi Immonen, Panu Lahtinen, Jaakko Pere

**Affiliations:** 1 Biocomposites and Processing, VTT Technical Research Centre of Finland, 33101 Tampere, Finland; 2 Biomass Processing Technologies, VTT Technical Research Centre of Finland, 02150 Espoo, Finland; panu.lahtinen@vtt.fi; 3 High Performance Fibre Products, VTT Technical Research Centre of Finland, 02150 Espoo, Finland; jaakko.pere@vtt.fi

**Keywords:** wood fibers, nanocellulose, composites, wood fiber composites, wood polymer composites, PLA

## Abstract

Thermoplastic composite materials containing wood fibers are gaining increasing interest in the manufacturing industry. One approach is to use nano- or micro-size cellulosic fibrils as additives and to improve the mechanical properties obtainable with only small fibril loadings by exploiting the high aspect ratio and surface area of nanocellulose. In this study, we used four different wood cellulose-based materials in a thermoplastic polylactide (PLA) matrix: cellulose nanofibrils produced from softwood kraft pulp (CNF) and dissolving pulp (CNFSD), enzymatically prepared high-consistency nanocellulose (HefCel) and microcellulose (MC) together with long alkyl chain dispersion-improving agents. We observed increased impact strength with HefCel and MC addition of 5% and increased tensile strength with CNF addition of 3%. The addition of a reactive dispersion agent, epoxy-modified linseed oil, was found to be favorable in combination with HefCel and MC.

## 1. Introduction

The growing awareness of environmental issues has directed a focus towards the use of more sustainable materials. Thermoplastic materials and thermoplastic composites are used as building materials in an increasing number of products due to their easy processing, free forms of design and the possibility to create products with lighter weight compared to metals or composites containing glass fiber [1]. Among polymer matrices, polylactide (PLA) is a good choice, being derived from renewable sources such as corn, sugar beet, sugar cane, cassava, but also from non-food cellulosic feedstocks such as bagasse, wheat straw or wood chips [2]. Those are natural glucose sources from which lactic acid, the monomer of PLA, can be produced by fermentation [3]. PLA has high strength and modulus compared to e.g., polyolefins, and is biodegradable, if only in industrial composting conditions. The disadvantages of PLA polymer are its low temperature resistance including low heat deflection temperature (HDT) (below 60 °C), low moisture resistance and low flexibility. However, for fiber composite materials PLA is an attractive matrix. When ligno-cellulosic fibers are added to PLA, they usually improve the tensile properties, but impact properties are weakened and the material becomes brittle if no coupling agents are added [4]. This is due to poor interaction between fiber and polymer, which has also been demonstrated in other studies [5,6,7].

Microcrystalline cellulose is an interesting reinforcement material for PLA, because it contains mainly crystalline cellulose and no weaker amorphous regions [8]. The crystallinity and low aspect ratio are expected to provide better dispersion to PLA than cellulose materials with a high aspect ratio. Microcrystalline celluloses are typically available in powder form and are much more easily applied in thermoplastic processes than nanocelluloses or cellulose pulp. The high specific surface area (>0.5 m^2^/g) and crystalline structure of microcellulose may also offer a greater reinforcing effect when compared to conventional cellulose fibers [9].

The use of nanocellulose in different forms and from different origins has been the focus in the composite research work of several authors [10,11]. Due to their advantageous mechanical properties and high surface area, nanocellulose fibers have good potential for utilization in load-bearing composites. The theoretical tensile strength values for nanocellulose crystals are in the range of 0.3–22 GPa and modulus values for a single cellulose nanofiber between 100 and 160 GPa. The specific surface area of nanocellulose is estimated to range between tens to hundreds of square meters per gram [12]. Improved composite properties have been demonstrated even with a very low degree of filling of nanocellulose, below 5% [13]. The most effective way for production of nanocellulose-reinforced composites is to use solvent casting, but from the manufacturing point of view thermoplastic processing is a more cost-effective method. Jonoobi et al. presented a combined thermoplastic extrusion process and solvent casting. PLA-CNF masterbatch was prepared by dissolving PLA in acetone-chloroform mixture and solvent-exchanged kenaf CNF from aqueous mixture to acetone, followed by mixing those two solutions and evaporating the solvent. This PLA-CNF blend was then mixed with PLA using extrusion, and injection molded to test specimens. The authors reported significant improvements in modulus and tensile strength of compounds, but also clearly visible aggregates of nanofibers in PLA [14].

Several approaches have been reported in literature to improve the compatibility of hydrophilic cellulose with hydrophobic PLA and to break the strong interaction between cellulose fibers. These include different fiber modifications (e.g., acetylation, esterification, silylation, silanization, oxidation, grafting, surfactants, coupling agents, plasticizers and physical modifications) [15,16,17,18,19]. Lu et al. modified cellulose nanofibrils to be more hydrophobic using amine-functionalization and gained improved strength properties for PLLA using solvent casting method and quite high nanomaterial addition (5–15%) [20]. Bulota et al. introduced acetylated microfibrillous cellulose to PLA with solvent casting method using fiber contents 2–20%. He had the best tensile strength results with fiber content over 10%. At 10% fiber content the Young’s modulus increased by approximately 15% and tensile strength remained the same. However, the strain at break increased from 8.4% to 76.1% with 5% fiber loading and DS 0.43 [21]. The review article from Oksman presents comprehensively several techniques for nanocellulose PLA composite manufacturing and mentions the use of plasticizers together with nanocellulose in addition to improve nanocellulose dispersion to PLA [22]. One group of additives also giving potential plasticizing effect is fiber de-bonders that enable cellulosic fibers to disperse more evenly on polymeric materials in a variety of absorbent products [23]. Our assumption was that a blend of non-ionic and cationic surfactants on cellulose fiber could improve the fiber dispersion in PLA, thus improving its strength properties. Another group of additives are epoxidized vegetable oils, which are bio-based plasticizer-stabilizers mainly used in PVC applications. They can also be used as plasticizers with other polymers such as PLA [24]. Miao et al. also prepared composites using only cellulose (paper) and epoxidized soy oil (ESO), which demonstrated the compatibility of ESO and cellulose [25]. For coupling they used a catalyzer, which is assumed not to be needed in this thermal molding process, due to the high temperature (>180 °C) in the process. When introduced on the surface of the fibers, ESO is assumed to improve fiber dispersion in PLA due to the long alkyl chain. The reaction between the OH group of cellulose and epoxies has been confirmed to proceed through the opening of the epoxide ring in acidic conditions, caused by residual moisture in cellulose [26]. Nanocellulose, having a high specific surface area, also has a large number of OH-groups enabling the reaction with epoxy groups even in lower temperature, which has been proven by Ansari et al. [27].

In this study, we prepared CNFs from three different wood-based raw materials and studied the effect of the raw material in thermoplastic PLA composites using thermoplastic compounding and injection molding as processing methods. There are certain challenges related to the thermoplastic processing and to achieving proper dispersion of the hydrophilic cellulosic material into the hydrophobic polymer. In order to minimize this effect we used two-stage compounding. For material comparison, we used microcellulose, which was easier to disperse into PLA than fibrous nanocelluloses. Our approach was also to treat the cellulose fibers with two different commercial long alkyl chain dispersion additives before compounding fibrils with PLA.

## 2. Materials and Methods

### 2.1. Polymer

Bio-based polylactide PLA 3052D (NatureWorks, Minnetonka, USA) was used as matrix polymer in this study. The polymer content in the studied materials was between 95% and 97%. PLA 3052D is a semi-crystalline polymer. It has melt flow index of 14 g/10 min (210 °C, 2.16 kg), specific gravity 1.24 and relative viscosity 3.3 [28]. It has an average molecular weight M_w_ 228.2 kg/mol and M_n_ 154.8 kg/mol determined in conjunction with this study by Virtanen et al. [29].

### 2.2. Nanocellulose Preparation

Cellulose nanofibrils (CNF) were produced using once dried bleached softwood kraft pulp from a Finnish pulp mill (MetsäFibre, Äänekoski, Finland) and softwood dissolving pulp from Domsjö Fabriker (CNFSD) (Örnsköldsvik, Sweden) followed by mechanical treatment with a high-shear grinder as described in the following. The pulps were first soaked at 1.8% consistency for one day and dispersed using a high shear Ystral Dispermix (Ystral, Markgräflerland, Germany) for 10 minutes at 2070 rpm. Suspension was then fed into a Masuko Supermasscolloider (Masuko Sangyo Co., Kawaguchi-city, Japan) type MKZA10-15J. The kraft pulp was ground with six passes and the dissolving pulp was ground seven passes in order to obtain the CNF. The rotation speed was fixed at 1500 rpm. The gap width was approximately 0.14–0.25 mm depending on the fibrillation cycle. The production yield of ground material was 95% based on mass balance calculation. The material was stored at +5 °C until used.

### 2.3. High-Consistency Nanocellulose Preparation

Bleached softwood pulp from a Finnish pulp mill (MetsäFibre, Äänekoski, Finland) was used as the raw material for producing CNF at high consistency (HefCel). The enzymatic treatment was carried out at a consistency of 25 w-% for 6 h at 70 °C using a two shaft sigma mixer (Jaygo Incorporated, NJ, USA) running at 25 rpm. The pulp batch size was 300 g on dry basis. After the treatment enzyme activity was stopped by increasing temperature of the mixer to 90 °C for 30 min. The fibrillated material was diluted with deionized water, filtered and washed thoroughly with deionized water. Finally, the fibrillated material was dewatered to a consistency of ~20% by filtration. The gravimetric yield of the fibrillated material was 90%. The material was stored at +4 °C until used.

### 2.4. Microcellulose

Powdery microcellulose (MC), Arbocel B600 was obtained from Rettenmeier and Söhne GmbH (Rosenberg, Germany). Typical topological polar surface area according to Chemical trading guide is 40.8 m^2^/g [30].

### 2.5. Nanocellulose Modification and Surface Treatments

In order to improve the dispersion of hydrophilic cellulosic fibers to hydrophobic PLA two different dispersion additives were used. Arosurf PA780, obtained from Evonik (Essen, Germany), is according to the manufacturer a fatty quarternary blend of non-ionic and cationic surfactants [31]. Referred to here as DA. It contains <20% imidazolium compounds, 2-C17-unsaturated-alkyl-1-(2-C18-unsaturated amidoethyl)-4,5-dihydro-N-methyl, Me sulfates [32]. Arosurf PA780 is a fiber de-bonder used in fluff pulp manufacturing [23]. In composites it was assumed mainly to increase hydrophobicity on fiber surface and to improve fiber dispersion to the polymer.

The second dispersion additive was epoxydized linseed oil Vikoflex 7190 from Arkema (Colombes, France), referred to here as VF (Vikoflex). It is recommended for plasticization and stabilization for polymers such as PVC and limits color formation during processing [33]. It has minimum 9.0% oxirane oxygen, capable of effecting a ring opening reaction in elevated temperature [34].

The introduction of both dispersing additives was carried by mixing the additives to CNF and HefCel water dispersions and MC powder in a dough mixer. DA was added to 20 w-% of fiber amount and VF to 10 w-% of fiber amount.

### 2.6. Drying

Before compounding with PLA HefCel and CNF were dried using a freeze-drying method. Freeze drying agglomerated fibrils to some extent, but it was the best available methods for this purpose. The water-containing slurry was frozen at −40 °C followed by freeze drying in a Supermodulyo 12K Freeze Dryer (Edwards High Vacuum International, Crowley, UK). The modified MC was oven dried at 50 °C overnight.

For plastic processing the PLA was dried in an oven at 50 °C overnight and nanomaterials were added directly from the freeze-drying process.

### 2.7. Plastic Processing

The compounding of materials to total cellulose contents of 3% or 5% in PLA was performed using a co-rotating Berstorff ZE 25x33D compounder (Berstorff GmbH, Hanover, Germany) and the compounds were injection molded to standard (ISO 527) dog bone shaped test pieces with an Engel ES 200/50 HL injection-molding machine (Engel Maschinenbau Geschellschaft m.b.H, Schwefberg, Austria). In order to ensure proper dispersion of fibrous material the compounding stage was performed twice. The reference PLA was also compounded as such before injection molding, in order to ensure the same thermal stress on materials. In compounding the temperature profile was from 165 °C in the feeding zone to 200 °C in the nozzle and the screw speed was 100 rpm. The temperature profile in injection molding was from the feed 180 °C to the nozzle 200 °C and the mold temperature was 25 °C.

### 2.8. Mechanical Testing

Tensile testing was performed according to ISO 527 using Instron 4505 Universal Tensile Tester (Instron Corp., Canton, MA, USA) mechanical test equipment. The results are the average of a minimum of five replicate samples with thickness 4 mm, total length 170 mm, in measurement point the test specimen length is 85 mm and width 10 mm.

Charpy impact strength was tested according to ISO 179 using unnotched samples flatwise and using a Charpy Ceast Resil 5.5 Impact Strength Machine (CEAST S.p.a., Torino, Italy). Sample size was 4 mm × 10 mm × 100 mm and the result is the average of 10 samples.

All the tested samples were conditioned at 23 °C and 50% relative humidity for a minimum of five days before testing.

### 2.9. SEM and Optical Microscopy

The morphologies of fibers and injection-molded samples were studied by scanning electron microscopy (SEM). The sample surface was coated with gold to prevent surface charging. In the case of injection-molded samples the scanning was made on cross-cut surfaces. Analyses were performed using JEOL JSM T100 (JEOL ltd., Tokyo, Japan) with a voltage of 25 kV.

Optical microscopy pictures were taken according to Kangas et al. [35].

## 3. Results and Discussion

### 3.1. Characterisation of Micro- and Nanocellulose Fibers

**Figure 1:**
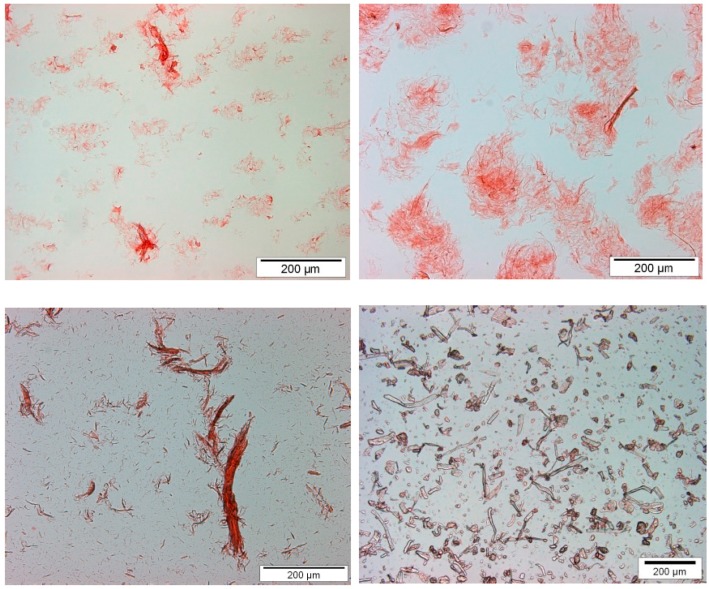
Images of fibrillated samples. CNF made of dissolving pulp CNFSD (**upper left**), kraft pulp CNF (**upper right**), HefCel CNF (**lower left**) and microfiber MC (**lower right**).

**Figure 2:**
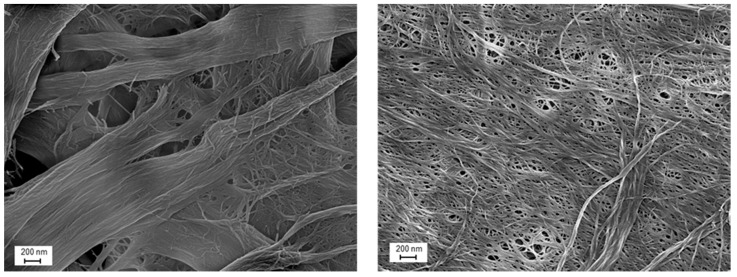
SEM images of fibrillated samples. HefCel CMF (**left**) and CNF kraft pulp (**right**).

Optical microscopy images of micro- and nanocelluloses are presented in Figure 1. According to the microscopic images only a few fibril bundles still existed in the CNF samples, but the amount of residual fibers was low. No clear differences were observed between CNF and CNFSD. MC appeared as round particles together with some long fibrous particles about 100 µm long (Figure 1 down right). The SEM images presented in Figure 2 provide a closer view of CNF and HefCel. Both CNF made of softwood pulp and HefCel appear as a network of slender fibrils and fibril aggregates. During the sample preparation HefCel had a high tendency to film formation, which partly covered the fibrillar network beneath. Morphological characteristics were evaluated based on optical microscopy and SEM. The average fiber dimensions of CNF, HefCel and MC are presented in Table 1.

### 3.2. Characterisation of Injection Moulded Test Bars

**Figure 3:**
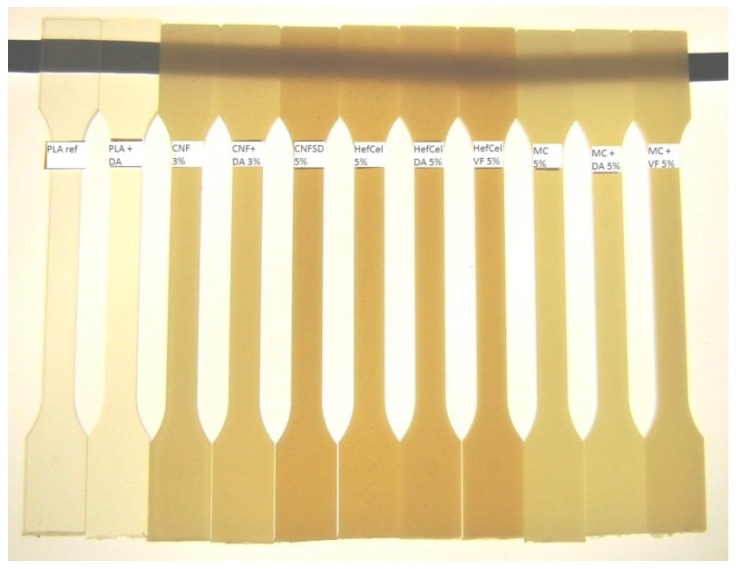
Injection molded test bars.

Injection molded test bars are shown in Figure 3. The dispersion of CNFs and HefCel into PLA was poorer than with MC and fibril aggregates, as observed in test bars containing CNF and HefCel. The addition of both the dispersion additives DA and VF improved the dispersion of all cellulosic materials to PLA. In composites containing MC the effect of dispersion agent cannot be seen due to the better dispersion capacity of MC as such, which may be due to its content of individual particles, without aggregates.

#### 3.2.1. SEM

**Figure 4:**
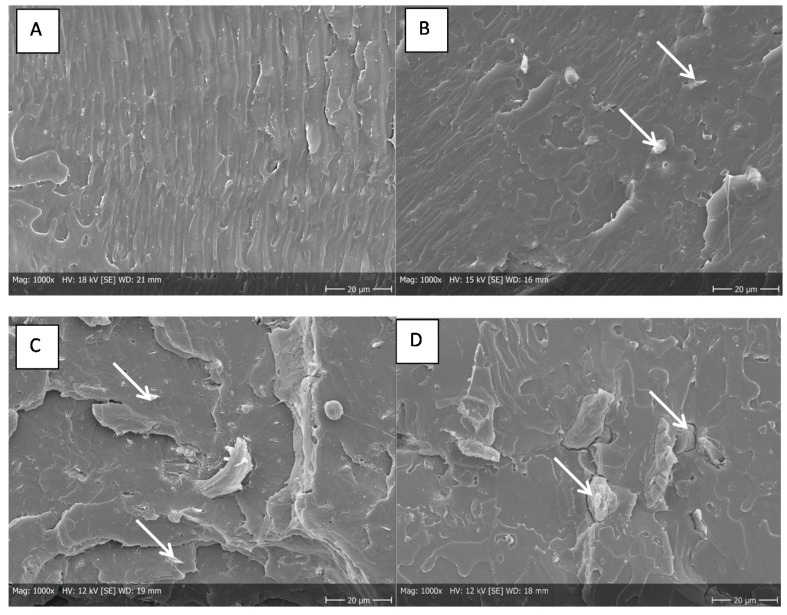
SEM image of injection molded test bars. (**A**) PLA matrix, (**B**) PLA with CNF, (**C**) PLA with HefCel CMF and (**D**) PLA with MC. Arrows are indicating fibers.

**Figure 5:**
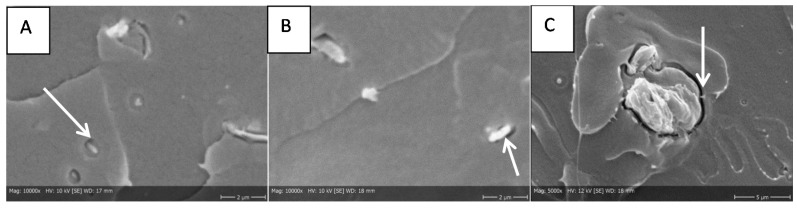
SEM image of injection molded test bars. (**A**) PLA with CNF, (**B**) PLA with HefCel CMF and (**C**) PLA with MC. Fiber loading 5%. Arrows are indicating a gap between the fiber and polymer.

Although the injection molded test bars contained visible cellulose aggregates, individual well-dispersed fibers were detected within the PLA matrix in SEM images (Figure 4). A closer look with a higher magnification shows a small orbicular area between fiber and polymer in all the composite samples indicating a poor connection between the PLA polymer matrix and the embedded cellulose fibrils and particles (Figure 5).

#### 3.2.2. Mechanical Results

**Figure 6:**
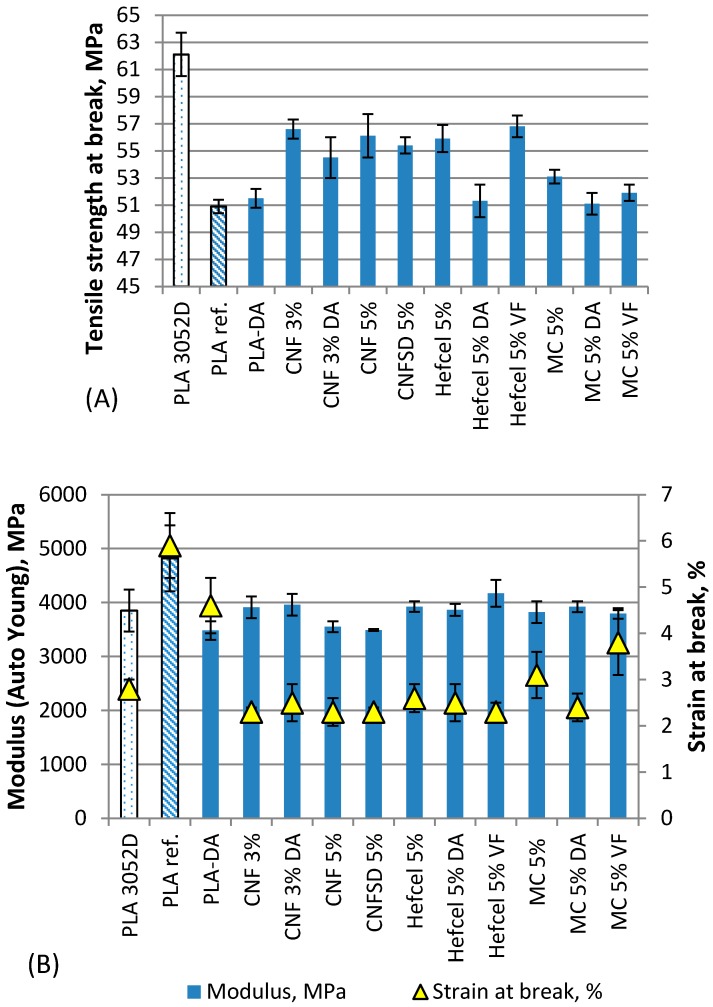
Tensile strength results (**A**), and modulus and strain at break (**B**) for injection molded PLA composites.

**Figure 7:**
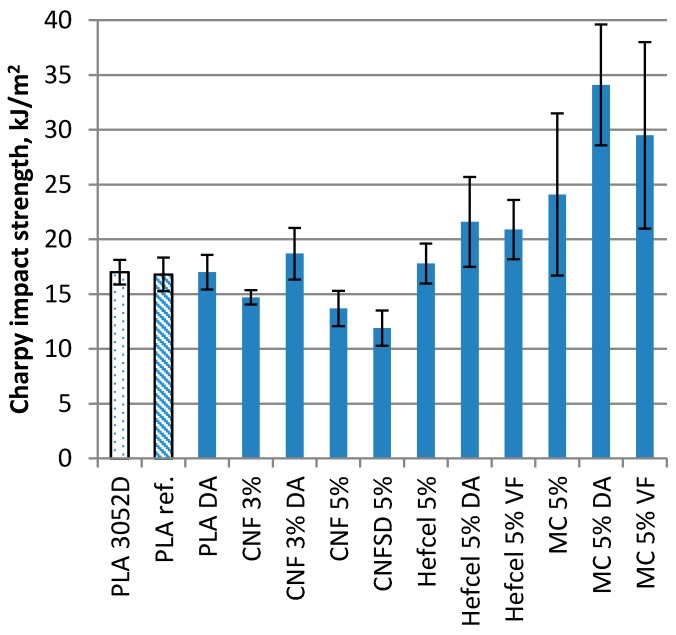
Charpy impact strength (unnotched) results for injection molded PLA composites with cellulosic fiber content of 3% or 5%.

Mechanical tests such as tensile strength and Charpy impact strength were performed for injection molded composite samples containing 3% or 5% cellulosic fibers in PLA. The tensile test results are presented in Figure 6 and the Charpy impact strength (unnotched) results in Figure 7. In the results, PLA 3052 is presented after injection molding and PLA ref., after going through the same compounding and injection molding processes as the fiber containing materials.

Reduced tensile strength and increased elongation and modulus can be seen for neat PLA after an additional compounding stage (Figure 6). The increase in modulus in twice compounded PLA ref compared to PLA 3052D may be related to increased crystallinity of PLA due to additional processing cycles. For example Tábi et al. observed increased crystallization in PLA sheets due to heat treatment [37]. The tensile strength reduction indicates PLA degradation due to additional thermal stress, which has also been found by other authors [38,39,40]. The effect of compounding on PLA 3052D molecular weight was determined in study by Virtanen et al. [29]. The same compounding method was used in this study and the current composite results can be compared to the PLA ref. and PLA-DA, which is PLA ref with DA. The addition of 1% of dispersion additive DA to PLA is able to plasticize the PLA and the effect can be seen as a 39% decrease in the modulus (PLA-DA).

When comparing the results without any dispersion additives, the PLA compounds with CNF, CNFSD, HefCel and MC gave very similar response in the tensile strength results; reduced strain at break, 19–27% decrease to modulus, but 4–11% increase in tensile strength at break value. Comparing the CNF addition in levels of 3% and 5% the CNF amount of 3% gave better tensile modulus (3910 MPa vs. 3550 MPa) and slightly better tensile strength value (56.6 MPa vs. 56.1 MPa). This can also be seen in the impact strength values (Figure 7) for CNF and CNFSD, which may indicate that the optimum value for CNF addition to PLA is below 5%. The lowest effect on tensile strength results was obtained with MC, which result is supported also by Bulota et al. [21]. The low optimum, below 5% of cellulosic nanomaterial to PLA, is supported by the results of other studies [19,41].

The dispersion additive DA appears to provide no additional improvements to tensile strength values with CNF, HefCel or MC compared to materials without dispersion additive. The presence of 3% CNF gave about 6% increase in tensile strength at break compared to PLA-DA without fibers (54.5 MPa vs. 51.5 MPa). 5% of HefCel-DA and MC-DA decreased the tensile strength by 4–6% compared to composite materials without DA. When the modulus of fiber materials with DA is compared to PLA-DA, there is an 11% to 13% increase in results with all fibers from 3480 MPa to over 3800 MPa, which indicates the stiffening effect of DA-containing materials due to the fibers. The same effect on the modulus was also observed when dispersion additive VF was used in combination with HefCel and MC. A 20% increase in modulus was observed with HefCel-VF and a 9% increase with MC-VF compared to PLA-DA. High modulus with HefCel-VF (4179 MPa) may indicate a reaction between fiber and polymer in addition to the improved dispersion presented in Figure 3. The strengthening of the dispersing additive VF containing HefCel can also be seen as an 8% higher tensile strength at break compared to HefCel-DA. The different effect of VF in HefCel and MC may be due to different surface area of fibers. HefCel has more surface area compared to MC thus providing more space for VF to react when applied on the fiber surface. The actual surface area of HefCel nanofibrillar material was not measured. Literature represents surface area for bleached softwood-based cellulose nanofibrils prepared with enzymatic method 304 m^2^/g [42] and without enzymes 195 m^2^/g [43]. According to SEM-pictures and fiber length, HefCel is nano-size material and is assumed to have larger specific surface area than MC (40.8 m^2^/g) [30]. Due to the small amount of fibers and additives in the polymer, also the actual reaction of VF between fibrils and polymer could not be verified by FTIR and only secondary indication through the mechanical results was used.

No significant difference between dissolving pulp CNF or kraft pulp CNF was observed in the tensile strength results in PLA composites. There was only a slight indication that kraft pulp CNF could be better for this purpose. Furthermore, no significant difference between CNF and HefCel was found in the tensile strength results.

The Charpy impact strength results in Figure 7 show no difference for neat PLA, after compounding (PLA ref.) or for PLA with DA. The addition of CNF (3% or 5%) and CNFSD (5%) caused 14–30% reduction in impact strength in comparison to PLA ref., which may be due to poor fibril dispersion. However the addition of pure HefCel increased the impact strength by 5% (from 16.8 to 17.8 kJ/m^2^) even with poor fibril dispersion. Pure MC addition had a clear positive effect on impact strength, as indicated by a 42% increase (from 16.8 to 24.1 kJ/m^2^), which may also have been partly due to better fiber dispersion of MC to PLA compared to nanofibers. The DA addition had a small positive effect on the CNF results probably due to improved dispersion. The same effect of DA can be seen for HefCel as a 27% increase in impact strength and for MC as a 100% increase in impact strength (from 17.0 to 34.1 kJ/m^2^). VF addition has a 17% positive effect on impact strength with HefCel (from 17.8 to 20.9 kJ/m^2^) and a 74% increase with MC (from 17.0 to 29.5 kJ/m^2^). The smaller effect of VF may also be due to the fact that DA addition to fiber was double compared to VF. Part of this impact strength increase may come from improved dispersion, and it appears that the aspect ratio also has an impact on dispersion effectiveness. The highest impact strength was obtained with MC with very good dispersion to PLA, and the second best was with HefCel with improved dispersion due to dispersing agents. DA improved the dispersion of CNF and gave slightly better impact strength results compared to composite without DA. However, there are still challenges to disperse the hydrophilic nanofibrillous material in hydrophobic PLA polymer.

The higher variance in impact strength results of MC containing composites may be due to their lower aspect ratio compared to nanomaterials and poor connection to the polymer matrix, which can be seen in Figure 5C.

## 4. Conclusions

In this study, cellulose nanofibrils (CNF and CNFSD) and high-consistency HefCel nanocellulose were prepared and their effects on PLA–cellulose composites were studied. Commercial microcellulose (MC) was used as comparison for nanocelluloses. The target was to explore the effects of these nanomaterials in thermoplastic PLA composites when used in small amounts of 3% to 5%. In the processing we used thermoplastic processes such as compounding and injection molding. We appreciated the challenges that may occur during the thermoplastic processing and in obtaining efficient dispersion of hydrophilic cellulosic material to hydrophobic polymer. A two-stage compounding process was used to improve the cellulosic material dispersion into polymer matrix. In addition, in order to improve the fiber dispersion to PLA, two different long alkyl chain dispersion-improving additives were introduced on the fiber before compounding with PLA. One of them, VF, was also able to cross-link fibers and polymers.

As a result it was found that the introduction of 3% to 5% cellulosic nanomaterial to PLA without dispersing additives provided no improvement in tensile strength at yield, modulus or elongation of PLA. However the tensile strength at break was improved 4%–11%. The best result without dispersing additives was obtained using 3% CNF in PLA. There was a 4%–28% reduction in the modulus compared to reference PLA. The highest reduction in modulus occurred when the dispersing additive DA was used without fibers. However when the dispersing additive was changed to a more reactive additive, VF, and the matrix was loaded with HefCel, the modulus increased by 20% compared to PLA-DA. This indicated toughening of fiber-PLA composites and a reaction between the fiber and the polymer matrix. A higher effect on modulus was observed with HefCel due to its greater surface area compared to MC.

The impact strength results showed 14–30% reduction when CNF and CNFSD were used without additives. No clear effect was observed with HefCel without additives. MC dispersed most easily on PLA and gave significantly better impact strength results compared to nanocellulose material. A 42% increase with MC was observed indicating the positive effect of good dispersion, but also indicating the effect of fiber dimensions on strength properties. The filler type fiber, MC, with its low aspect ratio, gave the best results for impact strength and even HefCel, with its wide variation of fiber lengths and fibrillation degree, gave better results than CNF. The improved dispersion due to the dispersion additives DA and VF improved impact strength with all the fibers. The reactive additive VF was able to increase impact strength when used at only 10 w-% on the fiber surface. This also indicates that the reactive additive VF is the more effective additive for cellulosic fiber in PLA composites than pure dispersing agents without the ability to cross-link fibers and polymers.

According to these results, there were no significant differences between dissolving pulp CNF and kraft pulp CNF in the tensile strength results in PLA composites. There was only slight indication that kraft pulp CNF could be better for this purpose. We observed that HefCel was easier to process and disperse in PLA than CNF, and that HefCel gave slightly better overall mechanical results than CNF. However, when the surface of nanocellulose can be modified for improved dispersion to polymers, there is more potential to further improve the PLA properties even at low dosages below 5%. This opens new potential uses for PLA in biomedical devices and implants in applications in which improved strength is needed. Especially in 3D-printing with PLA filament, there are challenges in the strength properties of thin-walled and structured parts due to the lack of pressure in manufacturing. Thus, improvements in inherent material strength are needed.

## Figures and Tables

**Table 1:** Fiber dimension of cellulosic materials used in PLA composites.

Fiber	Fiber Length, µm	Mean Fiber Width, nm
CNF/CNFSD	<10	15–40
HefCel	0.2–0.5	15–20
MC	Average 60 [36]	n.a.
